# Functional Analysis of *GarCIPK8* from *Gossypium aridum* Conferring Salt Stress Tolerance in *Gossypium hirsutum*

**DOI:** 10.3390/plants15213372

**Published:** 2026-11-01

**Authors:** Qi Guo, Jingqi Tao, Jinfeng Lv, Lili Du, Shucui Tao, Peng Xu, Jun Zhao, Liang Zhao, Jianguang Liu, Jianwen Xu, Jinhong Liu, Lin Jin, Xinlian Shen, Zhenzhen Xu

**Affiliations:** Key Laboratory of Cotton and Rapeseed (Nanjing), Ministry of Agriculture and Rural Affairs, Institute of Industrial Crops, Jiangsu Academy of Agricultural Sciences, Nanjing 210014, China; sevenguo86@163.com (Q.G.); tao2275010149@163.com (J.T.); jinfenglv66@163.com (J.L.); lili.du.2002@outlook.com (L.D.); taoshucui1234@163.com (S.T.); xupengjaas@126.com (P.X.); sxzhaojun88@aliyun.com (J.Z.); liangz@jaas.ac.cn (L.Z.); njljg@163.com (J.L.); gunman_xu@hotmail.com (J.X.); u3006988@connect.hku.hk (J.L.); xlshen68@126.com (X.S.)

**Keywords:** *GarCIPK8*, *G. aridum*, *G. hirsutum*, salt stress, antioxidant enzymes, ion homeostasis, transcriptome analysis

## Abstract

Soil salinization is a major abiotic stress factor restricting global agricultural production. The CBL-CIPK signaling network, comprising calcineurin B-like proteins (CBLs) and CBL-interacting protein kinases (CIPKs), plays a critical role in plant adaptation to salt stress. Here, we focused on *GarCIPK8*, a *CIPK* gene previously isolated from the diploid wild cotton *Gossypium aridum*, and generated its overexpressing transgenic lines in *G. hirsutum* through *Agrobacterium*-mediated transformation. Under 300 mmol/L NaCl treatment, transgenic plants showed markedly enhanced growth performance, with greater height and biomass accumulation than the wild type, and these differences increased with the duration of salt exposure, reaching a maximum at 30 d. This phenotypic advantage was associated with enhanced activities of superoxide dismutase (SOD) and peroxidase (POD), together with significantly reduced malondialdehyde (MDA) content. Specifically, at 3 d of salt stress, the transgenic lines exhibited significantly higher SOD activity than the wild type, while POD activity was markedly higher at both 1 d and 3 d. Ion content analysis revealed distinct distribution patterns between transgenic and wild-type plants. At 3 d and 7 d, transgenic roots accumulated significantly less Na^+^, whereas Na^+^ content in stems and leaves was substantially higher. In parallel, at 15 d, the transgenic lines showed significantly elevated root and stem K^+^ levels, as well as increased root Ca^2+^ content at the early stage of salt treatment (1 d and 3 d). Transcriptomic analysis further supported these phenotypic and physiological changes, as differentially expressed genes (DEGs) were significantly enriched in Gene Ontology (GO) terms related to antioxidant activity, peroxidase activity, and oxidative stress response, as well as in Kyoto Encyclopedia of Genes and Genomes (KEGG) pathways including calcium ion binding, metal ion transport, transporter activity, ABC transporters, and plant hormone signal transduction. These results demonstrate that *GarCIPK8* enhances salt tolerance in *G. hirsutum* by synergistically regulating ion homeostasis and antioxidant defense, providing a valuable genetic resource for cotton breeding.

## 1. Introduction

Soil salinization is a major abiotic stress factor that reduces global agricultural productivity by inducing severe cell dehydration and ion toxicity [1,2,3]. The CBL-CIPK signaling network, comprising calcineurin B-like proteins (CBLs) and CBL-interacting protein kinases (CIPKs), plays a critical role in plant responses to abiotic stresses, including salt, drought, and cold [4,5,6,7]. In this signaling network, CBLs act as calcium sensors that modulate downstream stress responses, while CIPKs are serine/threonine kinases that relay calcium signals [5]. The regulatory activity of this pathway is critically dependent on phosphorylation and protein–protein interactions [5,8,9,10]. Based on this mechanism, considerable progress has been made in understanding how the CBL-CIPK pathway enhances abiotic stress tolerance. Indeed, overexpression of *CBL* and *CIPK* genes has been shown to improve stress resistance in multiple plant species [11,12,13,14]. In cotton, the interaction between CBL2 and CIPK6 modulates sugar homeostasis by regulating the vacuolar membrane-localized sugar transporter TST2, thereby enhancing stress tolerance [15]. Notably, drought resistance in cotton is synergistically regulated by both negative and positive pathways, which are respectively governed by the GhCBL1A1-GhCIPK6D1 and GhCBL2A1-GhCIPK6D3 complexes [16].

Over the past few decades, numerous *CIPK* genes have been identified across diverse plant species [5]. Among the characterized members, *CIPK6*, *CIPK8*, *CIPK9*, *CIPK16*, *CIPK21*, and *CIPK24* have been reported to contribute critically to salt tolerance [14,15,17,18]. Notably, *CIPK24* (also known as *SOS2*) has been well characterized, acting as a core component of the CBL4–CIPK24–SOS1 module that facilitates Na^+^ extrusion and underpins adaptation to saline–alkaline environments [19,20,21]. Its close homolog, *CIPK8*, has drawn considerable attention for its key functions in abiotic stress responses [22,23]. In *Arabidopsis thaliana*, AtCIPK8 regulates the low-affinity phase of nitrate uptake [24,25] and forms a complex with CBL10 to activate the plasma membrane Na^+^/H^+^ antiporter SOS1, thereby enhancing salt tolerance [17]. The functional conservation of *CIPK8* is further supported by evidence from two independent species. In tomato, *SlCIPK8* enhances tolerance to cold, salt, and drought by elevating antioxidant enzyme activities, while overexpression of *CmCIPK8* in chrysanthemum similarly promotes salinity tolerance [26,27]. However, despite this evidence from model and horticultural species, the function of *CIPK8* in cotton, particularly in wild salt-tolerant cotton germplasm, and the physiological and transcriptional mechanisms through which it may improve salt tolerance remain largely unknown.

Cotton is a globally important crop that supplies natural fibers essential for the textile industry. Although it is commonly regarded as a pioneer crop for saline lands, salt stress still has a significant impact on its growth and development, leading to inhibited growth and reduced fiber production [28]. The domesticated allotetraploid species *Gossypium hirsutum* L. (2n = 4x = 52, (AD)_1_), known as Upland cotton, is globally cultivated for its high yield, moderate fiber quality, and wide environmental adaptability [29]. However, intensive artificial selection has narrowed the genetic diversity of modern Upland cotton cultivars, posing a challenge for further improvement of salt tolerance [30]. Therefore, exploring salt-tolerant germplasm resources and identifying superior salt tolerance genes are critical for deciphering the molecular responses to salt stress and accelerating molecular breeding in cotton. *G. aridum* (2n = 2x = 26), a diploid wild cotton species of the D_4_ group, is native to the Pacific coast of Mexico [30]. Our previous study showed that seeds of *G. aridum* achieved a germination rate of 92% under 1.2% NaCl treatment, indicating that this wild relative likely possesses beneficial alleles for salt tolerance that have been lost during domestication. Thus, *G. aridum* offers a valuable genetic resource for elucidating salt tolerance mechanisms, generating novel tolerant germplasm, and facilitating the development of improved cultivars [31]. To obtain a comprehensive overview of salt-responsive genes, we performed RNA-Seq analysis on roots and leaves of *G. aridum* under salt stress [32]. Among the differentially expressed genes (DEGs), *GarCIPK8*, encoding a *CIPK* family member, exhibited relatively high expression abundance [31,32]. However, whether *GarCIPK8* actually confers salt tolerance in cotton and which physiological and transcriptional pathways mediate its effects had not been determined. To address this knowledge gap, the present study aimed to: (i) overexpress *GarCIPK8* in *G. hirsutum* cv. R15 and evaluate its effects on salt tolerance through phenotypic, physiological, and biochemical measurements; and (ii) integrate transcriptomic analysis to identify the downstream pathways and candidate mechanisms through which *GarCIPK8* may enhance salt tolerance. This work provides a valuable genetic resource and mechanistic insights for the molecular breeding of salt-tolerant cotton varieties.

## 2. Results

### 2.1. Overexpression of GarCIPK8 Confers Enhanced Salt Tolerance in G. hirsutum

**Figure 1:**
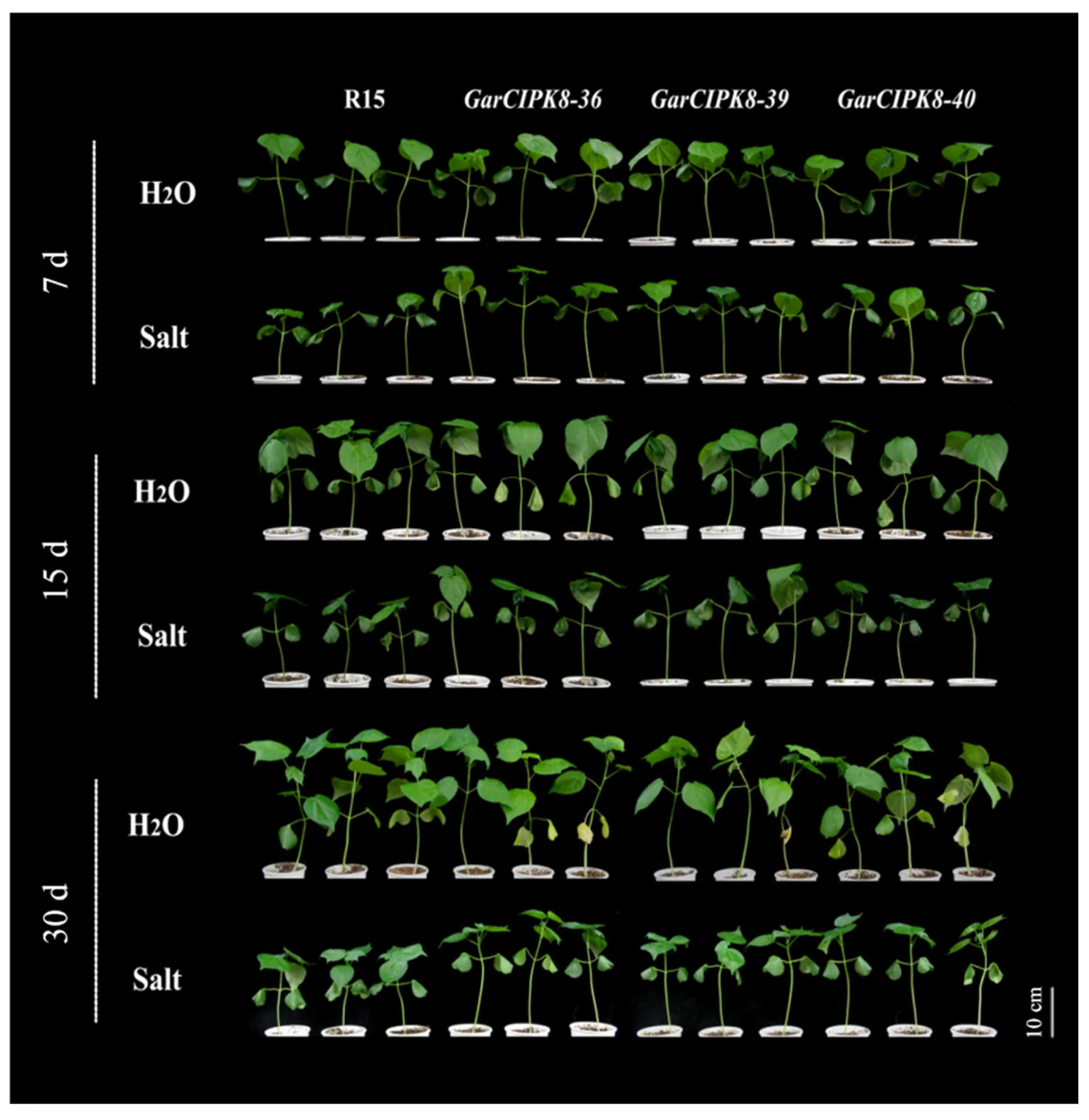
Phenotype of *GarCIPK8*-overexpressing lines and wild type under salt stress.

**Figure 2:**
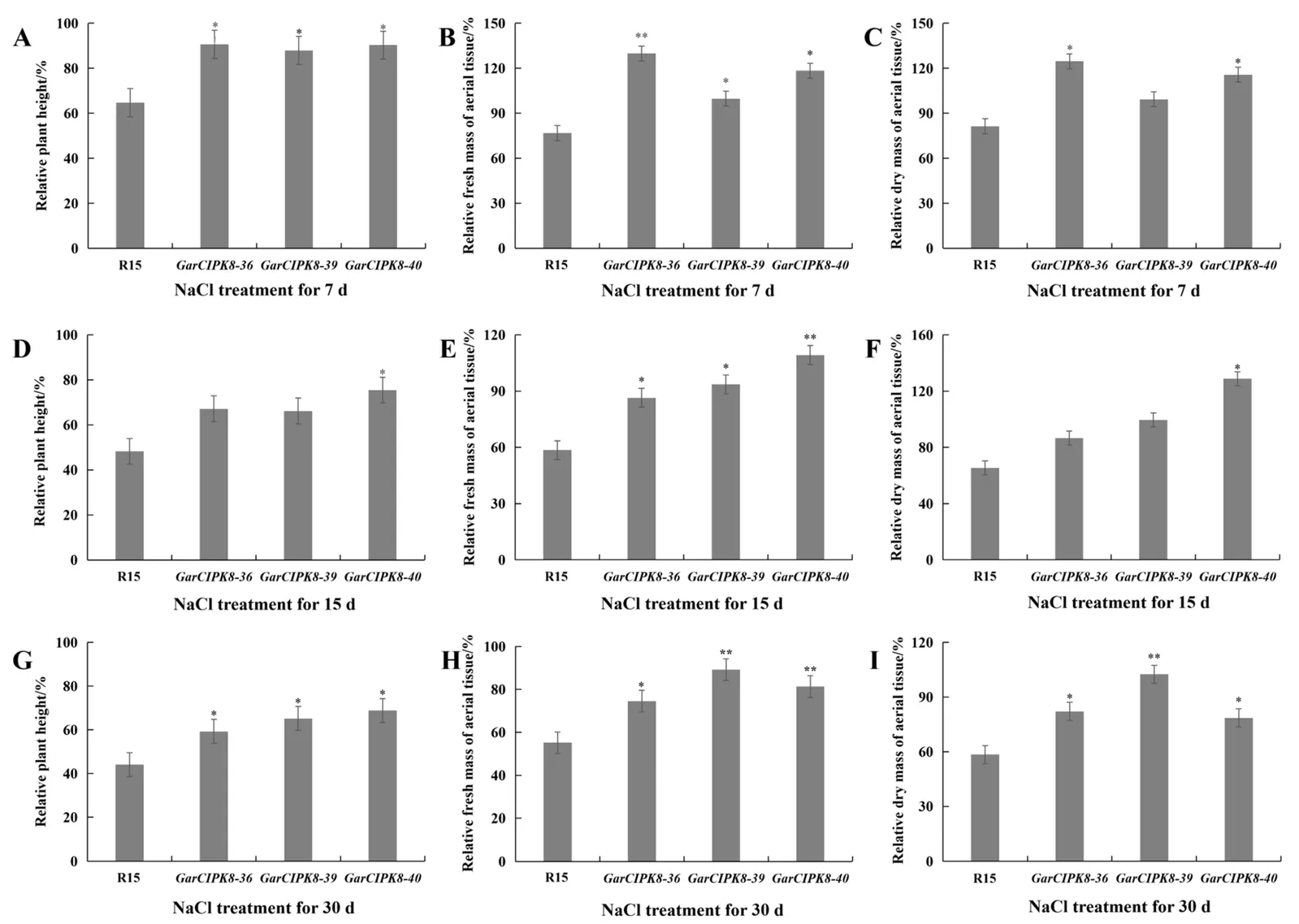
The biochemical indexes of *GarCIPK8*-overexpressing lines and wild type under salt stress. (**A**–**C**) Relative plant height, fresh mass of aerial tissues, and dry mass of aerial tissues at 7 d of NaCl treatment. (**D**–**F**) Relative plant height, fresh mass of aerial tissues, and dry mass of aerial tissues at 15 d of NaCl treatment. (**G**–**I**) Relative plant height, fresh mass of aerial tissues, and dry mass of aerial tissues at 30 d of NaCl treatment. The * and ** indicate statistical significance at the 0.05 and 0.01 probability levels, respectively.

To determine whether *GarCIPK8* enhances salt tolerance, we evaluated the phenotypic performance of three transgenic overexpression lines (*GarCIPK8-36*, *-39*, and *-40*) and the wild-type *G. hirsutum* cv. R15 at the seedling phase. All plants grew normally without salt stress but exhibited growth inhibition and leaf damage to varying degrees at 7, 15, and 30 d of 300 mmol/L NaCl treatment (Figure 1). The relative values of plant height, fresh weight, and dry weight of aerial tissues exhibited comparable temporal patterns under salt stress (Figure 2). At 7 d, the transgenic lines displayed significantly or highly significantly higher relative plant height and fresh weight of aerial tissues than the wild type (Figure 2A–C). At 15 d, their fresh weight of aerial tissues remained significantly or highly significantly higher (Figure 2D–F). By 30 d of salt stress, all three relative parameters were significantly or highly significantly greater in the transgenic lines compared with the control (Figure 2G–I). Notably, the extent of these differences increased with the duration of salt exposure, reaching the maximum at 30 d.

### 2.2. Overexpression of GarCIPK8 Improves Oxidative Stress Resistance in G. hirsutum

**Figure 3:**
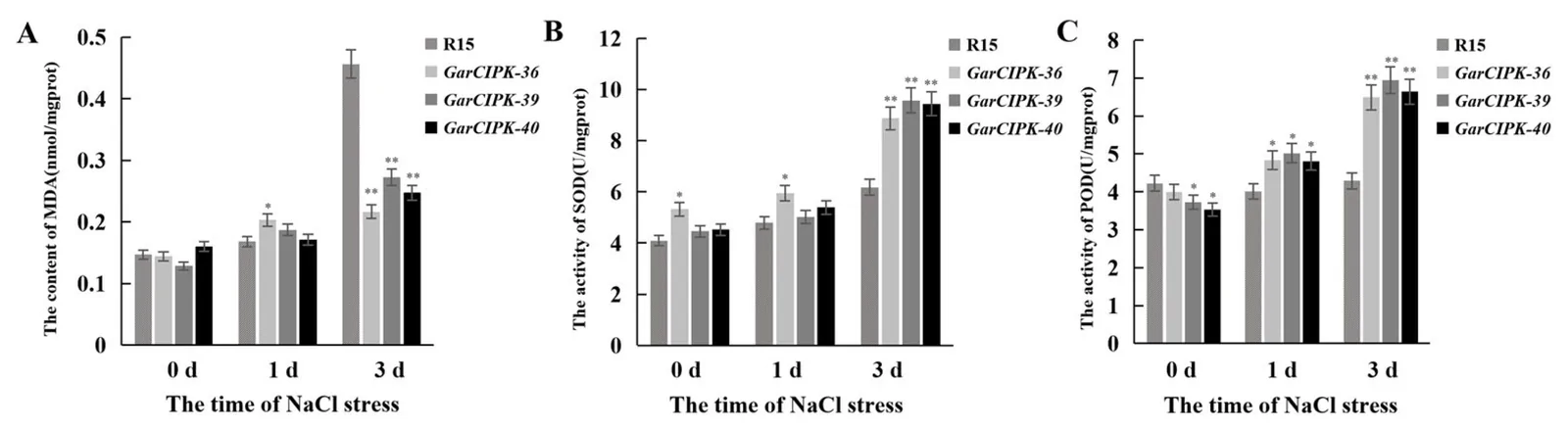
Physiological indexes of *GarCIPK8*-overexpressing lines and wild type under salt stress. (**A**) The content of MDA at 0, 1, and 3 d of NaCl treatment. (**B**,**C**) The activity of SOD and POD at 0, 1, and 3 d of NaCl treatment. The * and ** indicate statistical significance at the 0.05 and 0.01 probability levels, respectively.

To further characterize the physiological responses under salt stress, the malondialdehyde (MDA) content and the activities of peroxidase (POD) and superoxide dismutase (SOD) were measured in the second fully expanded leaves of the transgenic lines (*GarCIPK8-36*, *-39*, and *-40*) and *G. hirsutum* cv. R15. Transgenic lines appeared to alleviate salt-induced oxidative damage, as evidenced by the changes in MDA content and antioxidant enzyme activities (Figure 3). Following 300 mmol/L NaCl treatment, MDA levels increased in all plants and peaked at 3 d, at which point the transgenic lines showed a highly significant reduction in MDA content relative to the wild type (Figure 3A). This reduction in MDA content was consistent with the elevated activities of POD and SOD in the transgenic plants. Specifically, at 3 d of salt stress, the transgenic lines exhibited significantly higher SOD activity than the wild type, while POD activity was markedly higher at both 1 d and 3 d (Figure 3B,C).

### 2.3. Overexpression of GarCIPK8 Increased the Capacity of Ca^2+^\ K^+^\Na^+^ Uptake in G. hirsutum

**Figure 4:**
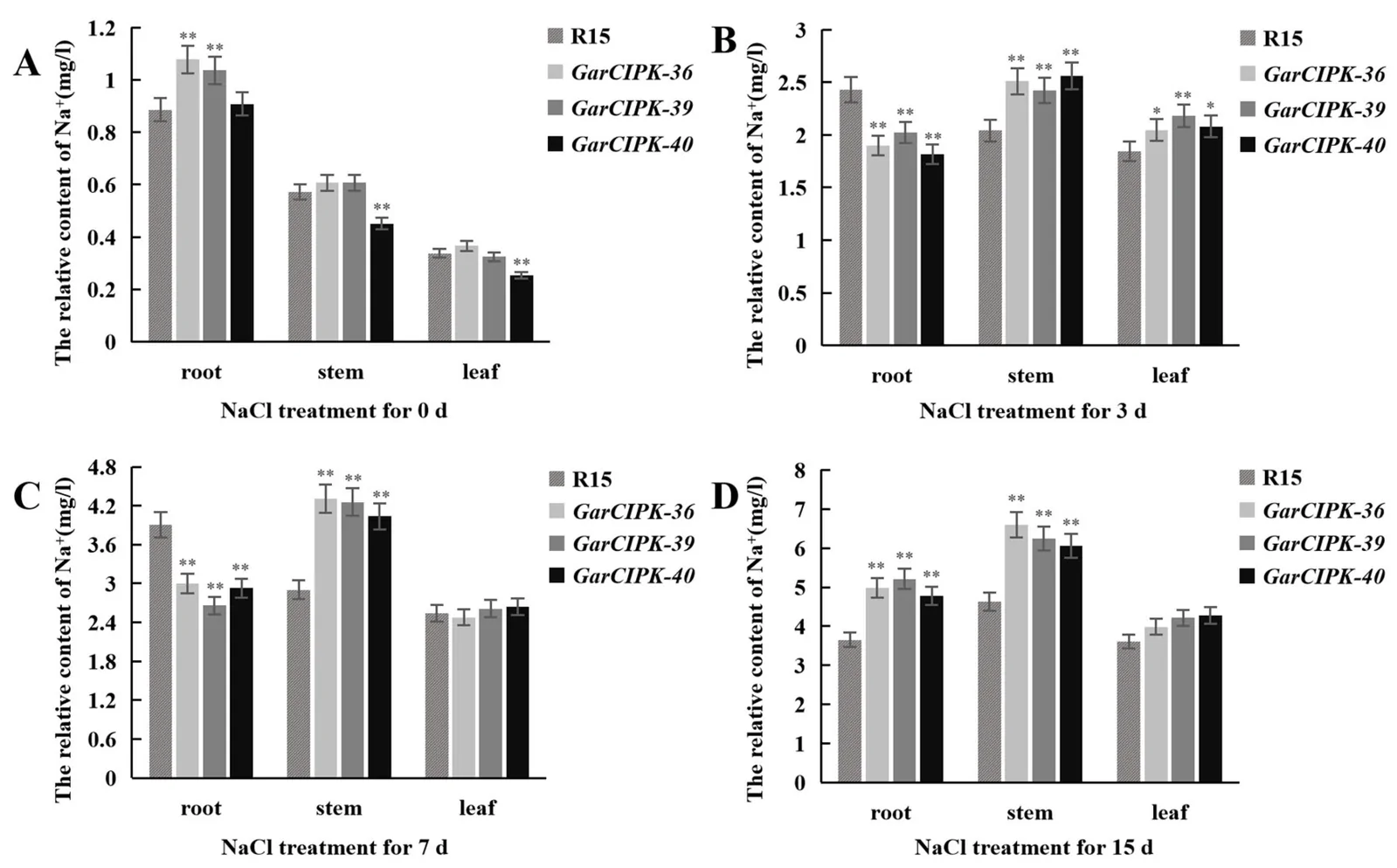
The content of Na^+^ in *GarCIPK8*-overexpressing lines and wild type under salt stress. (**A**–**D**) The relative Na^+^ content in root, stem, and leaf at 0, 3, 7, 15 d of NaCl treatment, respectively. The * and ** indicate statistical significance at the 0.05 and 0.01 probability levels, respectively.

To assess salt-induced ion accumulation, we quantified Na^+^ contents in the roots, stems, and leaves of three transgenic cotton lines (*GarCIPK8*-*36*, *-39*, and *-40*) and the wild type. At 0 d, Na^+^ distribution across all plants showed a consistent distribution pattern, with the highest levels in roots, followed by the stems and leaves (Figure 4A). After 3 d, the transgenic lines showed a highly significant reduction in root Na^+^ accumulation relative to the wild type, while Na^+^ levels in stems and leaves increased significantly or highly significantly (Figure 4B). This pattern persisted at 7 d, as root Na^+^ levels in the transgenic lines remained highly significantly lower and stem Na^+^ levels highly significantly higher than those in the wild type, while leaf Na^+^ concentrations remained unchanged (Figure 4C). At 15 d, both root and stem Na^+^ levels in transgenic plants had risen to highly significantly higher levels relative to the wild type, yet leaf Na^+^ still showed no significant difference (Figure 4D).

**Figure 5:**
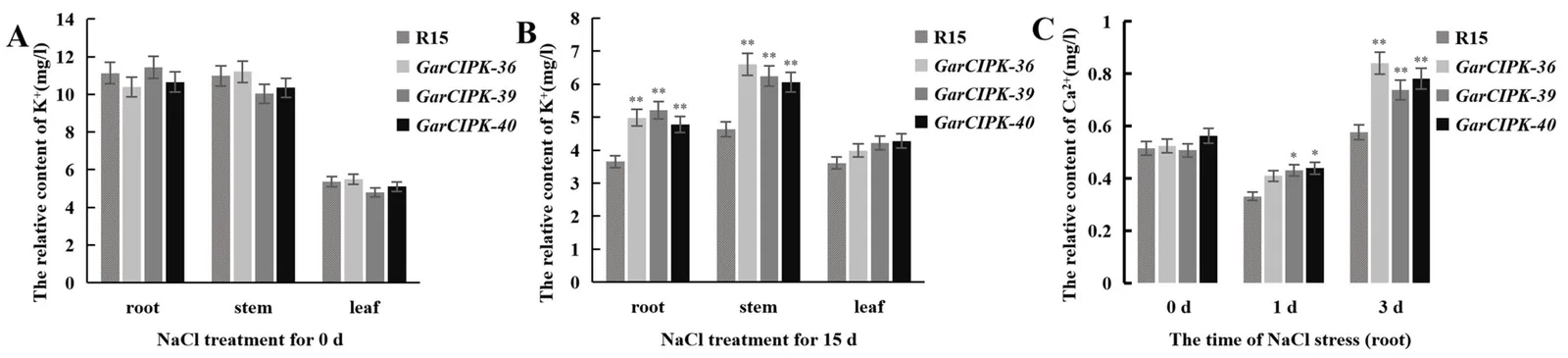
The content of K^+^ and Ca^+^ in *GarCIPK8*-overexpressing lines and wild type under salt stress. (**A**) Relative K^+^ content in different tissues (root, stem, and leaf) at 0 d of NaCl treatment. (**B**) Relative K^+^ content in different root, stem, and leaf) at 15 d of NaCl treatment. (**C**) Relative Ca^+^ content in roots at 0, 1, and 3 d of NaCl treatment. The * and ** indicate statistical significance at the 0.05 and 0.01 probability levels, respectively.

The contents of K^+^ and Ca^2+^ were measured to monitor the dynamic changes in ion transport during the stress period. At 0 h, there were no significant differences in K^+^ accumulation between the transgenic lines and wild type in any of the examined tissues, with the highest level detected in the roots, followed by stems and leaves (Figure 5A). After 15 days of salt exposure, the K^+^ concentrations in the roots and stems of the three transgenic cotton lines were highly significantly greater than those of the wild type (Figure 5B). For Ca^2+^ content, the transgenic lines exhibited significantly or highly significantly higher levels in roots than the wild type at both 1 d and 3 d, reaching the maximum at 3 d, whereas no such difference existed at 0 d (Figure 5C).

### 2.4. Gene Expression Profiles During Salt Stress

**Figure 6:**
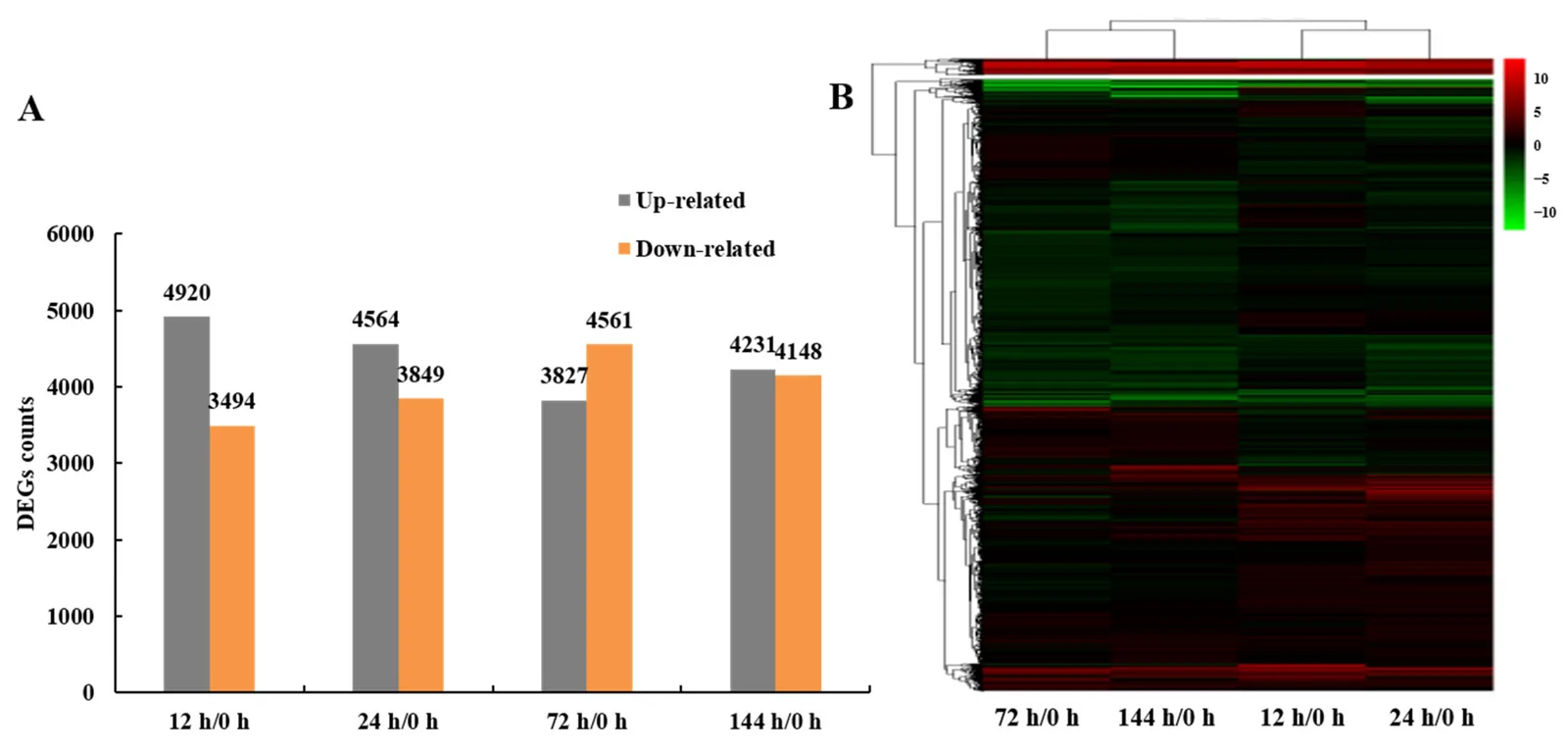
Counts (**A**) and hierarchical clustering (**B**) of differentially expressed genes (DEGs) in *GarCIPK8*-overexpressing lines under salt stress.

To characterize the transcriptional response under salt stress, we performed RNA–Seq analysis on root tissues of the *GarCIPK8-36* line exposed to salt stress for 12, 24, 72, and 144 h. Compared with 0 h, a total of 8414 (4920 up-regulated and 3494 down-regulated), 8413 (4564 up-regulated and 3849 down-regulated), 8388 (3827 and 4561 down-regulated), and 8379 (4231 up-regulated and 4148 down-regulated) DEGs were identified at 12, 24, 72, and 144 h of salt treatment, respectively (Figure 6A). The number of up-regulated genes peaked at 12 h, whereas the number of down-regulated genes reached its maximum at 72 h (Figure 6A).

Hierarchical clustering of DEGs based on their expression profiles revealed a temporal clustering of samples across different salt stress time points. Specifically, the 12 h and 24 h samples grouped together, representing a sustained regulation phase during the initial period of stress (Figure 6B). The other cluster, comprising the 72 h and 144 h samples, corresponds to a later stage characterized by rapid stress responses (Figure 6B). These results point to a time-dependent regulatory program, with distinct biological pathways and functional modules likely being activated at different phases of salt stress.

### 2.5. Gene Ontology (GO) Annotation and Enrichment Analysis

**Figure 7:**
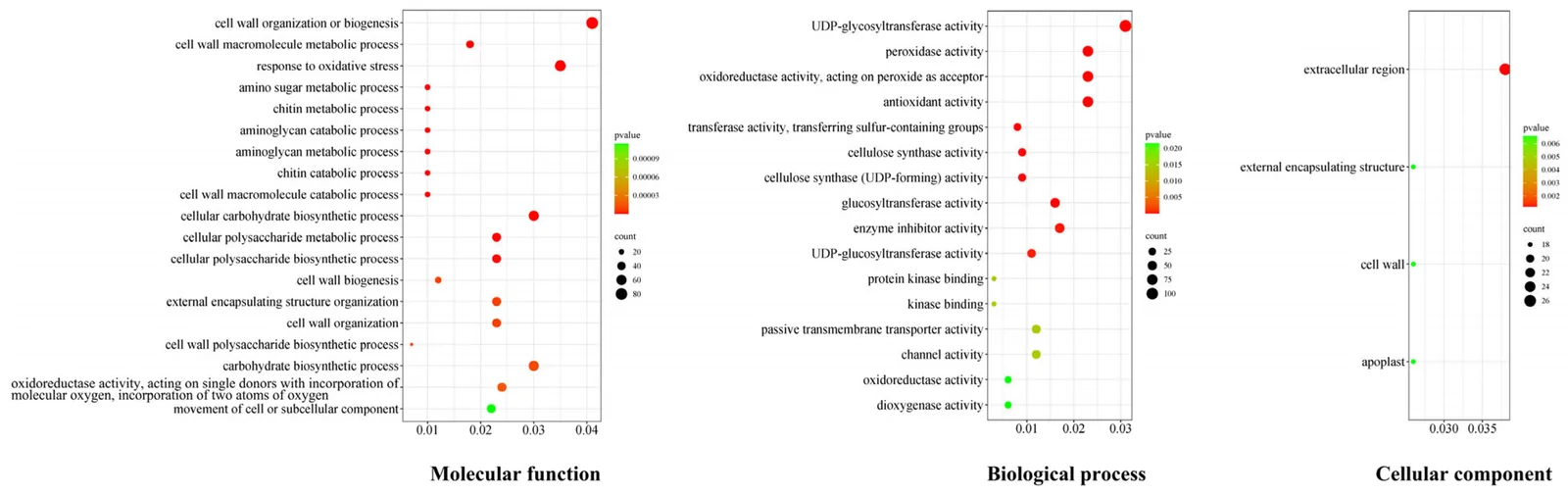
GO enrichment analysis of genes differentially expressed in *GarCIPK8*-overexpressing lines under salt stress. For each category, the significantly enriched GO terms are displayed, with the dot size representing the number of DEGs contained in each term and the color approaching red indicating increasing enrichment significance.

Gene Ontology (GO) annotation was performed to classify the 3679 annotated DEGs into 27 GO terms, which were distributed among biological process (BP, 16 terms), cellular component (CC, 1 term), and molecular function (MF, 10 terms) (Figure S2; Table S1). In the BP category, the DEGs were mainly assigned to metabolic process (GO:0008152), cellular process (GO:0009987), localization (GO:0051179), and response to stimulus (GO:0050896) (Figure S2; Table S1). Within the CC category, only one term, protein-containing complex (GO:0032991), was annotated (Figure 7; Table S1). For the MF category, the annotated terms included binding (GO:0005488), catalytic activity (GO:0003824), transporter activity (GO:0005215), transcription regulator activity (GO:0140110), and antioxidant activity (GO:0016209) (Figure S2; Table S1).

GO enrichment analysis (*p* ≤ 0.05) identified 616 DEGs that were significantly enriched in 92 GO terms, including 56 BP, 4 CC, and 32 MF terms (Table S2). In the BP category, the top enriched categories were cell wall organization or biogenesis (GO:0071554), cell wall macromolecule metabolic process (GO:0044036), and response to oxidative stress (GO:0006979). The MF category was significantly enriched in copper ion binding (GO:0005507), UDP-glycosyltransferase activity (GO:0008194), peroxidase activity (GO:0004601), oxidoreductase activity acting on peroxide as acceptor (GO:0016684), antioxidant activity (GO:0016209), and chitinase activity (GO:0004568). Only four GO terms were enriched in the CC category, including extracellular region (GO:0005576), cell wall (GO:0005618), external encapsulating structure (GO:0030312), and apoplast (GO:0048046) (Figure 7; Table S2).

### 2.6. Kyoto Encyclopedia of Genes and Genomes (KEGG) Annotation and Enrichment Analysis

A total of 1392 DEGs were assigned to six definite Kyoto Encyclopedia of Genes and Genomes (KEGG) pathway categories, including 1390 in metabolism, 159 in environmental information processing, 178 in genetic information processing, 108 in cellular processes, and 65 in organismal systems. Since a single DEG may be involved in multiple pathways, the total annotation count exceeded the number of uniquely mapped genes. Within the metabolism category, the distribution of pathway assignments was as follows: carbohydrate metabolism (261 DEGs), biosynthesis of other secondary metabolites (161 DEGs), amino acid metabolism (143 DEGs), lipid metabolism (113 DEGs), glycan biosynthesis and metabolism (93 DEGs), metabolism of terpenoids and polyketides (82 DEGs), metabolism of cofactors and vitamins (69 DEGs), energy metabolism (63 DEGs), metabolism of other amino acids (57 DEGs), and nucleotide metabolism (16 DEGs) (Figure S3; Table S3). For the genetic information processing group, the enriched pathways were folding, sorting and degradation (88 DEGs), translation (63 DEGs), replication and repair (44 DEGs), chromosome (19 DEGs), transcription (16 DEGs), and information processing in viruses (5 DEGs) (Figure S3; Table S3). The environmental information processing group comprised only two pathways: signal transduction (ko04016, ko04070, and ko04075) and membrane transport (ko02010), containing 169 and 40 DEGs, respectively (Figure S3; Table S3). For the cellular processes category, the two represented pathways were transport and catabolism (76 DEGs) and cell motility (69 DEGs). In the organismal systems category, the only pathway identified was environmental adaptation, which included 87 DEGs (Figure S3; Table S3).

**Figure 8:**
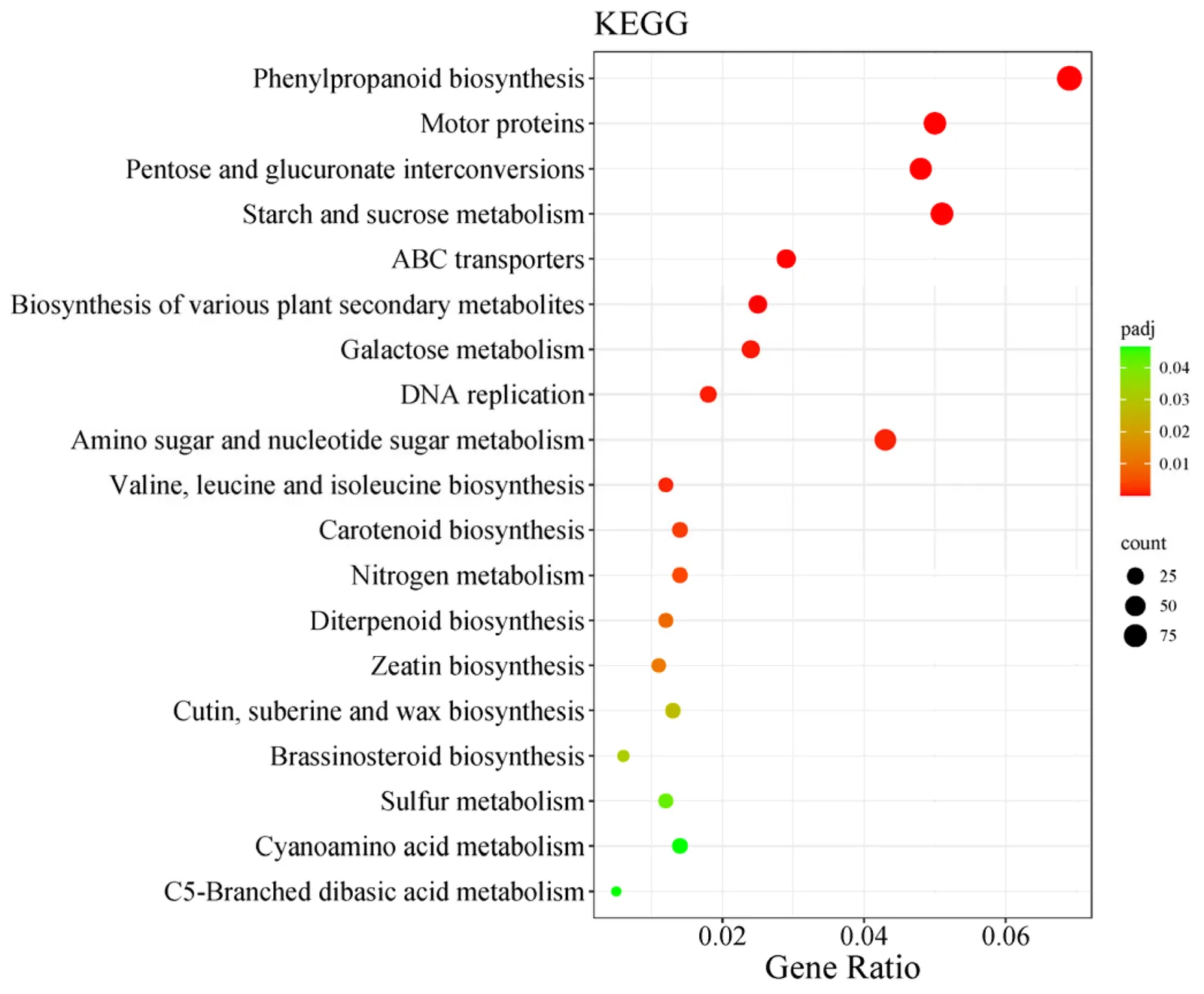
Scatter diagram of KEGG pathway enrichments for genes differentially expressed in *GarCIPK8*-overexpressing lines under salt stress.

We also constructed a scatter plot to identify significantly enriched KEGG pathways. A total of 19 KEGG pathways were significantly enriched among 564 DEGs, with the top pathways being phenylpropanoid biosynthesis (ko00940), motor proteins (ko04814), pentose and glucuronate interconversions (ko00040), starch and sucrose metabolism (ko00500), ABC transporters (ko02010), biosynthesis of various plant secondary metabolites (ko00999), galactose metabolism (ko00052), DNA replication (ko03030), amino sugar and nucleotide sugar metabolism (ko00520), valine, leucine and isoleucine biosynthesis (ko00290), carotenoid biosynthesis (ko00906), nitrogen metabolism (ko00910), diterpenoid biosynthesis (ko00904), zeatin biosynthesis (ko00908), cutin, suberine and wax biosynthesis (ko00073), brassinosteroid biosynthesis (ko00905), sulfur metabolism (ko00920), cyanoamino acid metabolism (ko00460), and C5-branched dibasic acid metabolism (ko00660) (Figure 8; Table S4).

### 2.7. Validation of RNA-Seq Data Using qRT-PCR

**Figure 9:**
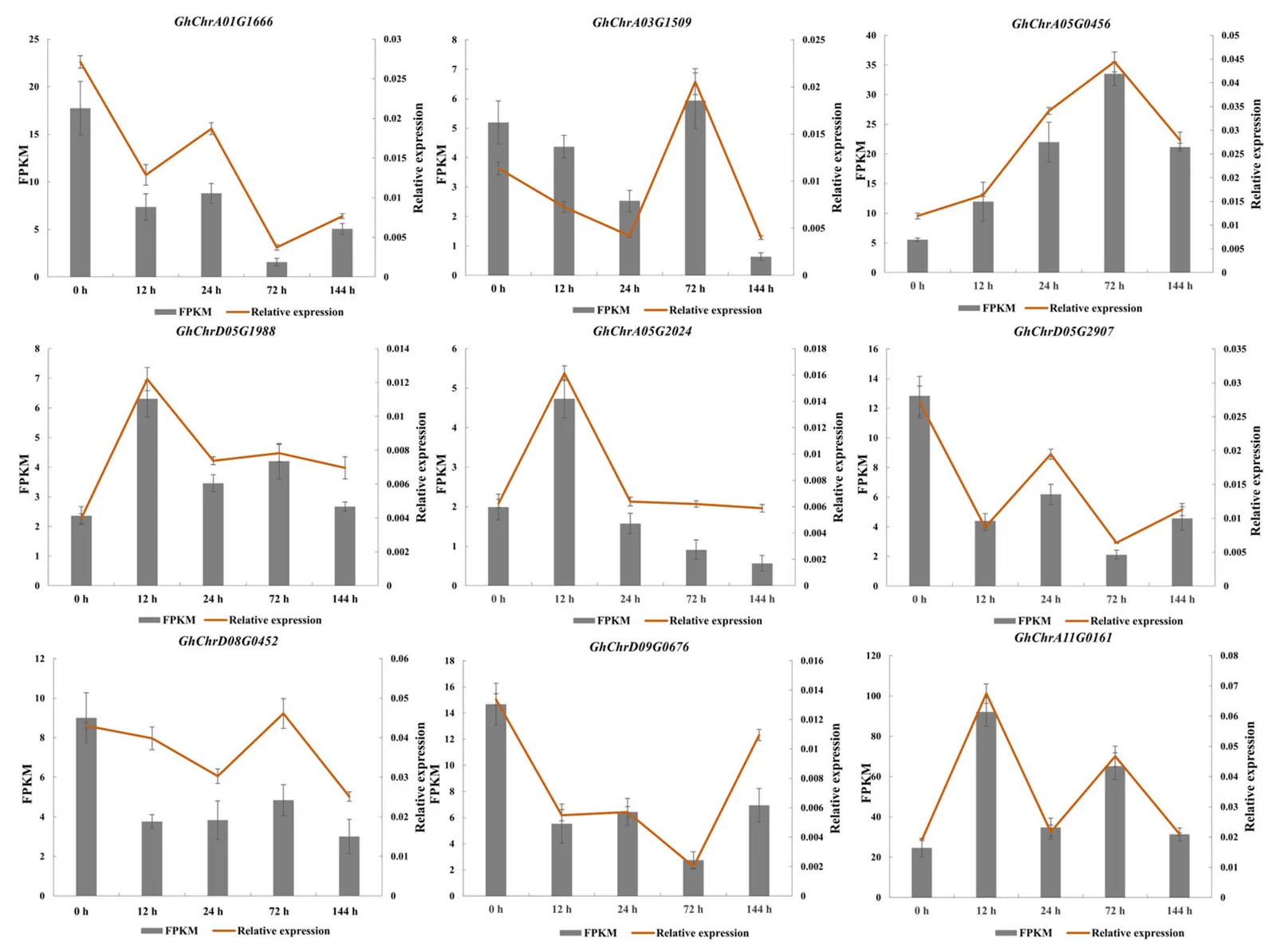
**The** qRT-PCR validation of nine candidate gene expression patterns identified via RNA-Seq. The left *y*-axis corresponds to the RNA-Seq FPKM values, and the right *y*-axis indicates the relative expression determined via qRT-PCR.

Nine candidate genes potentially involved in salt tolerance were selected for quantitative real-time PCR (qRT-PCR) analysis based on their expression profiles in the transcriptome data and the functional annotations of their *Arabidopsis* orthologs. These nine genes included two peroxidase genes (*GhChrA03G1509* and *GhChrA05G2024*), two glycosyl hydrolase family genes (*GhChrD05G2907* and *GhChrD09G0676*), an auxin-responsive protein gene (*GhChrD05G1988*), a phosphate uridylyltransferase gene (*GhChrD08G0452*), a glucuronoxylan 4-O-methyltransferase gene (*GhChrA01G1666*), and two calcineurin B-like protein genes (*GhChrA11G0161* and *GhChrA05G0456*). The expression patterns of these nine genes determined via qRT-PCR were consistent with the RNA-seq data (Figure 9 and Table S5).

## 3. Discussion

### 3.1. Overexpression of GarCIPK8 Confers Salt Tolerance by Enhancing the Antioxidant Defense Mechanism in G. hirsutum

The CBL-CIPK regulatory network, which operates downstream of calcium (Ca^2+^) signaling, has long been implicated in plant adaptation to abiotic stresses such as salinity, potassium deficiency, drought, and cold [23,33,34]. In the present study, *GarCIPK8*-overexpressing cotton plants exhibited significantly better growth than wild-type plants under salt stress at the seedling stage, as evidenced by increased plant height and biomass accumulation. These phenotypic improvements were substantiated by both physiological responses and transcriptomic analyses. At 1 d and 3 d of salt exposure, transgenic lines exhibited higher POD and SOD activities and lower MDA content. Transcriptome analysis at 12 h and 24 h revealed that DEGs were significantly enriched in GO categories linked to antioxidant activity, peroxidase activity, and oxidative stress response, while KEGG pathway analysis highlighted up-regulation of phenylpropanoid biosynthesis. These findings suggest that *GarCIPK8* mediates a rapid oxidative stress defense response, likely by up-regulating the transcription of genes involved in ROS scavenging [33].

### 3.2. GarCIPK8 Enhances Salt Tolerance in G. hirsutum by Coordinately Regulating Na^+^ Sequestration and K^+^ Homeostasis

Ion homeostasis is a critical determinant of plant salt tolerance [4,5,34,35]. In the present study, *GarCIPK8*-overexpressing lines displayed distinct ion distribution profiles, characterized by lower root Na^+^ accumulation and higher stem/leaf Na^+^ content relative to the wild type at 3 d and 7 d of salt stress. These lines also maintained higher K^+^ levels in both roots and shoots after 15 days of salt treatment. While these observations suggest that *GarCIPK8* may facilitate root-to-shoot Na^+^ translocation and vacuolar sequestration to mitigate ionic toxicity in roots, our current dataset lacks direct mechanistic validation. Accordingly, we present this as a working hypothesis, and definitive confirmation will require future experimental evidence, including Na^+^ flux measurements, xylem sap analysis, and CBL-CIPK interaction assays.

Transcriptomic analysis further substantiated these physiological observations. DEGs were significantly enriched in GO terms associated with calcium ion binding, metal ion transport, and transporter activity, and KEGG analysis revealed up-regulation of ABC transporters and plant hormone signal transduction pathways. Temporal transcriptome data showed that genes related to ion transport were modulated at early stages (12 h and 24 h), paralleling the early Ca^2+^ response. Given the well-characterized functions of the CBL-CIPK signaling module, we propose that *GarCIPK8* likely interacts with *CBL* to activate downstream transporters such as SOS1 and NHX [20,36,37], thereby coordinating Na^+^ exclusion and vacuolar sequestration, while simultaneously transducing stress signals via Ca^2+^ signaling. These results support a model in which *GarCIPK8* serves as an early regulator of ion homeostasis, coordinating multiple transport processes to alleviate salt-induced ionic imbalance.

### 3.3. GarCIPK8 Confers Significant Potential for Salt Tolerance Improvement in G. hirsutum

Overexpression of *GarCIPK8* not only substantially promoted seedling growth in *G. hirsutum* but also conferred comprehensive regulatory capacity for salt tolerance at both physiological and transcriptional levels. In comparison with previously characterized genes such as *CIPK6* and *CIPK24* [16,18,37], our results demonstrate that *GarCIPK8* effectively coordinates ion transport and antioxidant defense. Furthermore, our data suggest a potential role for *GarCIPK8* in promoting Na^+^ vacuolar sequestration. Given the natural adaptation of the donor species *G. aridum* to saline environments, the stable expression and heritable transmission of *GarCIPK8* in *G. hirsutum* highlight its considerable potential for salt tolerance breeding. For future perspectives, CRISPR-based gene editing could be used to further dissect its functional domains, and pyramiding *GarCIPK8* with other salt tolerance genes may facilitate the development of elite cotton germplasm with improved salinity tolerance and broader adaptability.

## 4. Materials and Methods

### 4.1. Expression Vector Construction and Transformation

A 1350 bp *CIPK* gene, designated *GarCIPK8*, was isolated from *G. aridum* by means of in silico cloning in our previous work [30]. Here, the coding sequence of *GarCIPK8* was cloned into the pCAMBIA2301 expression vector downstream of the *cauliflower mosaic virus* (CaMV) 35S promoter, with *BamHI* and *KpnI* sites used for insertion. The primers used for amplification were F: 5′CGCGGATCCGATGGTGGTGAGAAAAGTAGGGAAGTA3′ and R: 5′GGGGTACCCAGGTCAACGTCTTTTACTCCTAGA3′. The resulting recombinant plasmid pCAMBIA2301-*GarCIPK8*, harboring the *neomycin phosphotransferase II* (*NptII*) gene, was transformed into *G. hirsutum* cv. R15 by means of *Agrobacterium*-mediated transformation. Following kanamycin resistance selection and PCR screening, a total of 11 independent T_0_ transgenic plants were obtained, representing seven transgenic lines (Figure S1A). The three selected independent transgenic lines were advanced to the T_5_ generation through successive self-pollination. At each generation (T_1_–T_5_), genomic PCR was performed to genotype the plants and evaluate the segregation ratios of the selectable marker. T_5_ lines whose progeny consistently tested positive (100% positive rate) were confirmed as homozygous. Consequently, the three T_5_ lines used in this study originated from independent transformation events. We confirm that the three lines used represent truly independent transformation events. Based on qRT-PCR analysis, three positive transgenic lines (namely, *GarCIPK8-36*, -*39*, and -*40*) with high expression levels of *GarCIPK8* were selected for further analysis (Figure S1B).

### 4.2. RNA Isolation and Semi-Quantitative RT-PCR

Total RNA was extracted from leaves of the transgenic lines with a plant RNA purification kit (Omega, Beijing, China) according to the manufacturer’s instructions. Subsequently, 2 micrograms of total RNA per reaction was reverse transcribed into cDNA using RT-Perfect Real Time Master Mix (TaKaRa, Dalian, China). Then, 2 μg of total RNA was reverse-transcribed to cDNA using the RT-Perfect Real Time Master Mix (TaKaRa, Dalian, China). The resulting cDNA was used for RT-PCR with gene-specific primers for GarCIPK8 (F: 5′-GAAAGCCTTGCCATGAAAGTCCTC-3′; R: 5′-GCTTCCGTTTCACTAAGACGACCTT-3′). The endogenous actin gene of G. hirsutum (F: 5′-GAGGCATTCCACCTGACCAAC-3′; R: 5′-CTTGACCTTCTTCTTCTTGTGCTTG-3′) was used as an internal control. The PCR products were electrophoresed on 1.2% agarose gels and photographed with an imaging system (Bioshine GelX 1520, Shanghai, China).

### 4.3. Evaluation of Salt Tolerance in Transgenic Cotton

To evaluate salt tolerance at the seedling stage, one seed of the wild type or each transgenic line was sown per paper cup (7 cm in diameter and 8 cm in height) and watered with 70 mL of water. Plants were grown in a growth chamber at 26 ± 2 °C under a 16 h light/8 h dark photoperiod. At the two-leaf stage, plants grown in soil were subjected to salt stress by means of irrigation with 75 mL of 300 mmol/L NaCl solution, whereas control plants were irrigated with an equal volume of distilled water [38]. Each treatment was performed with three biological replicates, each containing 10 plants. Plant height, fresh weight, and dry weight of aerial tissues were measured as biomass parameters at 7, 15, and 30 d after salt treatment.

### 4.4. Measurement of Physiological Indexes

To measure physiological parameters, leaves were collected from salt-treated and control plants of all lines, immediately frozen in liquid nitrogen, and stored at −70 °C for further analysis. The activities of POD and SOD, as well as MDA content, were assayed using commercial assay kits (Jiancheng Bioengineering Institute, Nanjing, China).

For ion content determination, plants were harvested at 3, 7 and 15 d of salt treatment. Five plants from each transgenic line and the wild type were individually divided into root, stem, and leaf tissues. After oven-drying at 80 °C for 24 h to constant weight, 0.2 g aliquots of the samples were transferred to digestion tubes and mixed with 5 mL of a sulfuric acid–hydrogen peroxide mixture (30%, *v*/*v*). Digestion was performed in a microwave system (CME MARS 6 CLASSIC) at 195 °C for 25 min. The digests were then analyzed for Na^+^, K^+^, and Ca^2+^ concentrations by means of atomic absorption spectrophotometry (PerkinElmer PinAAcle 900T, Waltham, MA, USA), with all measurements performed in triplicate.

### 4.5. Library Preparation for RNA-Seq

Total RNA was extracted from the roots of transgenic line *GarCIPK8-36* and wild-type plants at 0, 12, 24, 72, and 144 h after 300 mmol/L NaCl treatment. For each material and time point, three biological replicates (individual plants) were used. RNA integrity and concentration were assessed with the RNA Nano 6000 Assay Kit on an Agilent Bioanalyzer 2100 system (Agilent Technologies, Santa Clara, CA, USA). Sequencing libraries were prepared using the Illumina TruSeq Stranded RNA Library Preparation Kit, pooled according to effective concentration and target data volume, and then sequenced on the DNBSEQ-T7 platform (MGI, Shenzhen, China) to generate 150 bp paired-end reads.

### 4.6. Differential Expression Analysis

Raw reads were filtered with FastQC (v0.11.9) to remove adapters and low-quality bases, and the clean reads were aligned to the *G. hirsutum* reference genome (ZJU-improved_v2.1_a1) [28] using HISAT2 (v2.0.5) [39]^.^ Gene expression was quantified as read counts by featureCounts (v1.5.0p3) [40]. Differential expression analysis was conducted with DESeq2 (v1.20.0) [41] by comparing each time point (12, 24, 72, and 144 h) to the control (0 h). DEGs were identified using thresholds of |log_2_(fold change)| ≥ 1 and adjusted *p*-value (*P*adj) ≤ 0.05, with the false discovery rate (FDR) controlled using the Benjamini–Hochberg method.

### 4.7. GO and KEGG Annotation and Enrichment Analysis

All DEGs were mapped to the GO [40] and KEGG [41] databases, and the number of DEGs corresponding to each GO term and KEGG pathway was quantified. To identify significantly enriched terms and pathways, the DEGs were considered as the foreground list, with all annotated genes in the genome serving as the background. Statistical significance was assessed using the hypergeometric distribution algorithm (Phyper), and the resulting *p*-values were adjusted for multiple testing by controlling the FDR value.

### 4.8. Validation of RNA-Seq Data via qRT-PCR

To validate the RNA-seq data, the expression levels of nine selected genes were confirmed by means of qRT-PCR. The endogenous cotton *histone-3* gene (GenBank accession no. *AF024716*) was used as the reference gene to normalize cDNA amounts. Gene-specific primers were designed using SnapGene software (version 6.0.2). The qRT-PCR was performed using ChamQ Universal SYBR qPCR Master Mix (Vazyme, Nanjing, China) on a QuantStudio 5 Real-Time PCR System (Applied Biosystems, Foster City, CA USA). Relative transcription levels were calculated using the 2^−ΔCt^ method, where ΔCt is the difference in Ct values between the target gene and the reference gene (*histone-3*).

### 4.9. Statistical Analysis

All experiments were performed with three biological replicates. Data were analyzed using SPSS 26.0 (IBM, Armonk, NY, USA,) and presented as means ± standard deviation (SD). Student’s *t*-test was used for comparisons between two groups. A *p*-value < 0.05 and *p*-value < 0.01 were considered statistically and highly significant, respectively.

## 5. Conclusions

In this study, we demonstrate that *GarCIPK8*, a *CIPK* gene from the diploid wild cotton species *G. aridum*, acts as a positive regulator of salt tolerance in *G. hirsutum*. Overexpression of this gene markedly improved plant growth under 300 mmol/L NaCl treatment in *G. hirsutum*. Physiological and ion content analyses revealed that *GarCIPK8* confers salt tolerance through the synergistic regulation of antioxidant defense and ion homeostasis, as evidenced by increased antioxidant enzyme activities, reduced oxidative damage, and time- and tissue-dependent Na^+^ and K^+^ distribution patterns coupled with enhanced Ca^2+^ signaling under salt stress. Transcriptome profiling further supported these findings, showing coordinated enrichment of DEGs involved in oxidative stress responses, ion transport, and calcium signaling. These results show that *GarCIPK8* enhances salt tolerance in *G. hirsutum* by integrating antioxidant and ion homeostatic mechanisms, making it a promising gene for cotton breeding.

## Data Availability

All of the raw sequencing data generated in the current study have been deposited in the BioProject database of the NCBI under accession number PRJNA697836.

## Supplementary Materials

The following supporting information can be downloaded at: https://www.mdpi.com/article/10.3390/plants15213372/s1, Figure S1: The PCR (A) and qRT-PCR (B) detection of *GarCIPK8* in T_5_ transgenic homozygous lines; Figure S2: GO annotation distribution of genes differentially expressed in *GarCIPK8*-overesressing lines under salt stress; Figure S3: KEGG annotation distribution of genes differentially expressed in *GarCIPK8*-overesressing lines under salt stress; Table S1: The GO annotation for differentially expressed genes in *GarCIPK8*-overesressing cotton lines under salt stress; Table S2: The enrichment GO terms for differentially expressed genes in *GarCIPK8*-overesressing cotton lines under salt stress; Table S3: The KEGG annotation for differentially expressed genes in *GarCIPK8*-overesressing cotton lines under salt stress; Table S4: The enrichment KEGG terms for differentially expressed genes in *GarCIPK8*-overesressing cotton lines under salt stress; Table S5: The qRT-PCR Primers for nine Genes.
