# Supplementary material for: Functional Analysis of GarCIPK8 from Gossypium aridum Conferring Salt Stress Tolerance in Gossypium hirsutum
Source: Plants (Basel). 2026 Nov 1;15(21):3372. doi: 10.3390/plants15213372 (PMC13765565; doi:10.3390/plants15213372)
Supplement: Supplementary file 1 [file plants-15-03372-s001.zip › Supplementary Materials.pdf]

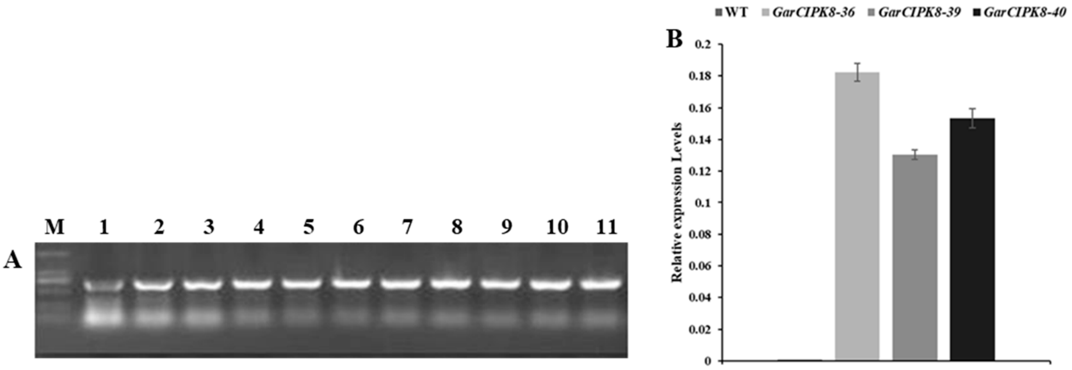

Figure S1. **The PCR (A) and qRT-PCR (B) detection of *GarCIPK8* in T_5 transgenic homozygous lines.** M: DL5000 Marker.

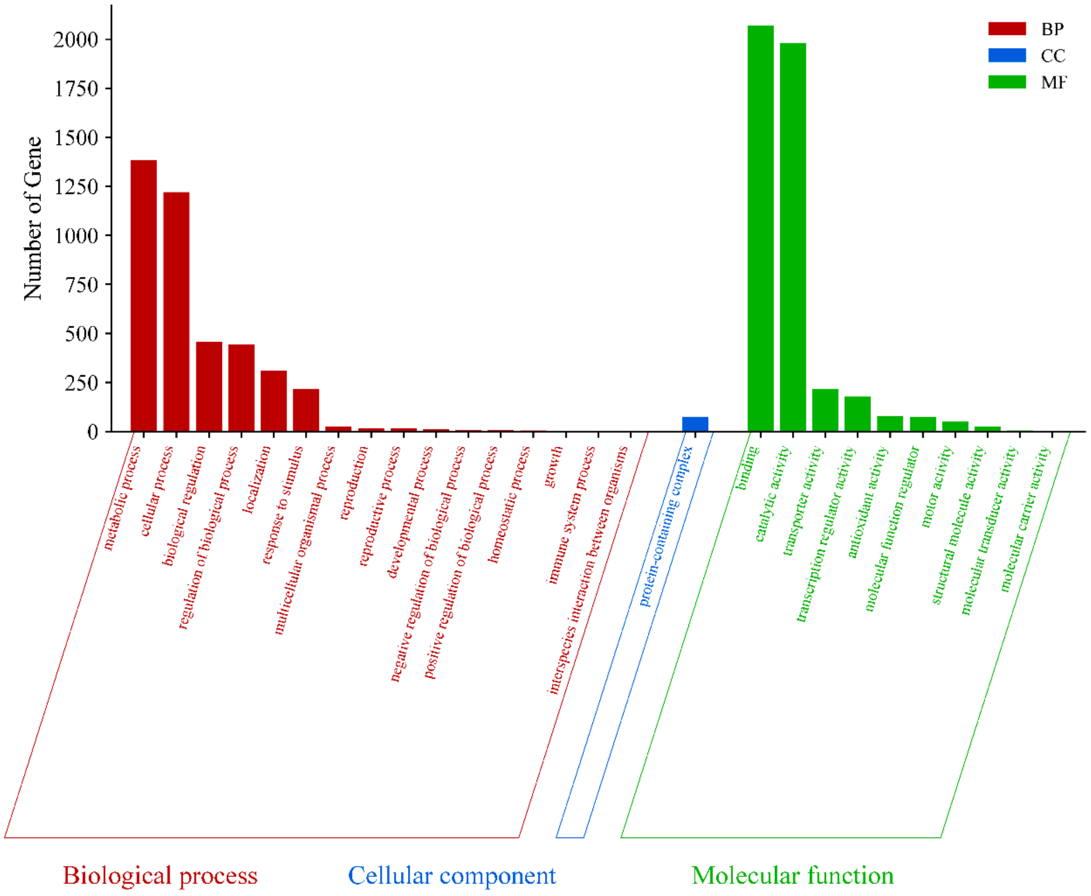

Figure S2. GO annotation distribution of genes differentially expressed in *GarCIPK8*-overesressing lines under salt stress. Histograms represent the functional distribution; the left y-axis shows the number of annotated genes, and the right y-axis represents the percentage of total genes.

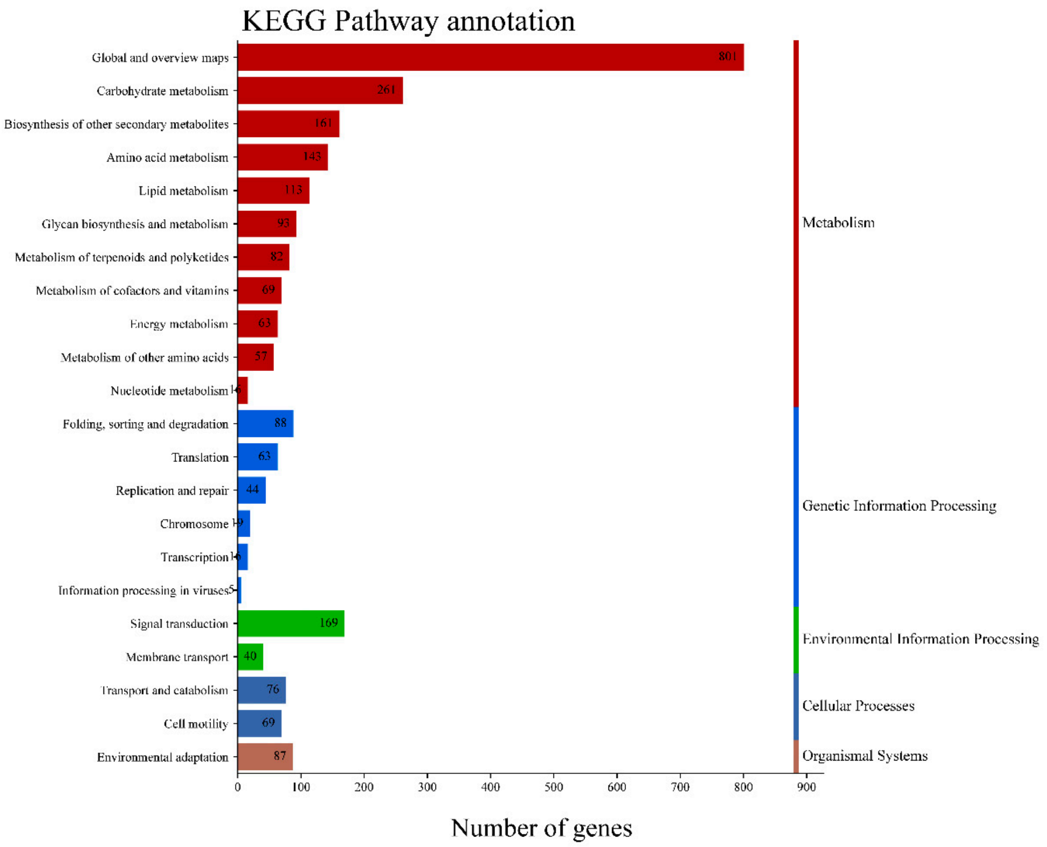

Figure S3. KEGG annotation distribution of genes differentially expressed in *GarCIPK8*-overesressing lines under salt stress. The Rich Factor is defined as the ratio of the number of DEGs annotated in a given pathway term to the total number of genes annotated in that term, with larger values corresponding to stronger enrichment.
